# Instantaneous Rigor Causing Trismus During Cardiac Arrest Requiring Emergency Surgical Airway: A Case Report

**DOI:** 10.7759/cureus.101914

**Published:** 2026-01-20

**Authors:** Takshak Shankar, Nidhi Kaeley, Ajay Kumar Mallapu, Sarath S Nair, Gaurav Juneja

**Affiliations:** 1 Emergency Medicine, All India Institute of Medical Sciences, Rishikesh, Rishikesh, IND

**Keywords:** airway management, cadaveric spasm, cardiac arrest, cricothyrotomy, trismus

## Abstract

Instantaneous rigor, which is also known as cadaveric spasm, is a rare condition characterized by muscle rigidity occurring immediately at the moment of death, cardiac arrest, or peri-cardiac arrest. When it involves the temporomandibular joint, it can result in sudden trismus and create an unanticipated airway emergency during resuscitation.

An elderly female in her late 60s was brought to the emergency department with altered sensorium and shortness of breath following a massive episode of hematemesis, along with progressive abdominal distension. She had underlying hepatitis C-related chronic liver disease. On arrival, she was critically ill with a Glasgow Coma Scale score of E1V1M2, unrecordable blood pressure, bradycardia, and signs of shock. During initial resuscitation, the patient developed cardiac arrest with asystole as the initial rhythm. Cardiopulmonary resuscitation was initiated; however, immediately following arrest, severe trismus developed, rendering bag-mask ventilation or orotracheal intubation impossible. An emergency front-of-neck access was promptly performed using surgical cricothyrotomy, and the airway was successfully secured within five minutes. Despite 30 minutes of advanced life support, return of spontaneous circulation was not achieved. Laboratory findings revealed severe anemia, coagulopathy, metabolic acidosis, hyperkalemia, and acute kidney injury.

Instantaneous rigor is a rare but critical cause of sudden airway failure during cardiac arrest. Early recognition and prompt transition to a surgical airway are essential to avoid delays in ventilation. Emergency physicians should remain proficient in emergency surgical airway techniques to manage such unpredictable scenarios effectively.

## Introduction

Instantaneous rigor, also called cadaveric spasm, is often an uncommon entity characterized by sudden and pronounced muscle rigidity occurring at the moment of death or during severe hypoxia-induced cardiac arrest [1-3]. Unlike rigor mortis, which develops gradually, this rigidity occurs immediately and may involve the muscles of mastication, resulting in trismus and creating significant difficulty in airway management [4,5]. Because airway control is a critical component of cardiac arrest management, alternative airway strategies may be required in such situations [6]. We report a case of an elderly female who developed severe trismus following cardiac arrest, necessitating emergency surgical airway management.

## Case presentation

An elderly female in her late 60s presented to the ED with shortness of breath and altered sensorium following a massive episode of hematemesis (approximately 500 mL). She also reported progressively increasing abdominal distension over the preceding four days. She was a known case of hepatitis C virus (HCV)-related chronic liver disease and had defaulted from treatment.

On arrival at the ED, the patient appeared critically ill, pale, and obtunded. Her Glasgow Coma Scale (GCS) score was E1V1M2. Airway examination revealed copious blood-tinged frothy oral secretions, which were promptly suctioned. A detailed assessment of mouth opening was limited because of reduced responsiveness. Breathing was labored and shallow, with a respiratory rate of 28 breaths/minute.

Circulatory assessment revealed bradycardia with a pulse rate of 58 beats/minute, absent peripheral pulses, unrecordable blood pressure, and clinical features of shock. Capillary refill time was prolonged (>3 seconds), and the extremities were cold and clammy. Pupillary examination was normal and equal bilaterally. Point-of-care capillary blood glucose was 235 mg/dL. A focused neurological examination did not reveal focal deficits; however, assessment was limited due to the low GCS score.

Abdominal examination revealed gross abdominal distension consistent with tense ascites; further palpation was deferred because of hemodynamic instability. Clinical stigmata of chronic liver disease, including icterus and bilateral pedal edema, were noted. A comprehensive systemic examination could not be completed due to rapid clinical deterioration. Relevant investigations are presented in Table 1.

**Table 1:** Relevant investigations performed at an outside facility prior to presentation. AST: aspartate aminotransferase; ALT: alanine aminotransferase; INR: international normalized ratio; PaCO2: partial pressure of carbon dioxide; HCO3: bicarbonate.

Investigation	Result	Reference range
Hemoglobin	6.8 g/dL	12–16 g/dL
Platelet count	78 ×10⁹/L	150–450 ×10⁹/L
Total bilirubin	4.2 mg/dL	0.3–1.2 mg/dL
AST/ALT	112/68 IU/L	<40 IU/L
INR	2.1	<1.2
Serum albumin	2.4 g/dL	3.5–5.0 g/dL
Serum sodium	128 mmol/L	135–145 mmol/L
Serum potassium	5.8 mmol/L	3.5–5.0 mmol/L
Blood urea nitrogen	62 mg/dL	7–20 mg/dL
Serum creatinine	2.3 mg/dL	0.6–1.2 mg/dL
pH	7.18	7.35–7.45
PaCO2	29 mmHg	35–45 mmHg
HCO3	11 mmol/L	22–26 mmol/L
Lactate	6.7 mmol/L	<2 mmol/L
Ultrasound of the abdomen	Shrunken nodular liver, gross ascites, splenomegaly	-

While preparations for airway management were underway, the patient developed cardiac arrest with an initial rhythm of asystole. High-quality cardiopulmonary resuscitation (CPR) was initiated. Immediately following the arrest, severe trismus was observed (instantaneous rigor), making bag-mask ventilation or orotracheal intubation impossible. The team proceeded with front-of-neck access (FONA) using a surgical cricothyrotomy and successfully secured the airway with a size 6 endotracheal tube within five minutes of initiating CPR.

Despite best efforts and continued advanced life support for 30 minutes, she could not be revived, and the patient was declared deceased.

## Discussion

Instantaneous rigor is a rare phenomenon characterized by immediate muscle stiffening at the time of death or cardiac arrest [1]. It is distinct from rigor mortis, which typically develops two to six hours after death [1]. The underlying mechanism is thought to involve rapid depletion of adenosine triphosphate (ATP), which is required for muscle relaxation. When ATP is abruptly depleted, actin-myosin cross-bridges remain interlinked, resulting in sustained muscle contraction [2,3]. Although the biochemical basis is similar to rigor mortis, the timing differentiates the instantaneous rigor.

Several clinical conditions have been associated with instantaneous rigor, including severe stress or trauma, drowning, electrocution, opioid intoxication, seizures, metabolic acidosis, and hyperkalemia [1-5]. These factors were relevant in our patient, who presented with hemorrhagic shock, metabolic acidosis (pH: 7.18; partial pressure of carbon dioxide (pCO2): 29 mmHg; bicarbonate: 11 mmol/L; lactate: 6.7 mmol/L), hyperkalemia (5.8 mmol/L), and acute kidney injury (blood urea nitrogen (BUN): 62 mg/dl; serum creatinine: 2.3 mg/dl) in the setting of decompensated chronic liver disease.

In such circumstances, early recognition and prompt transition to surgical airway management are essential to avoid delays in ventilation and resuscitation [7]. Emergency surgical airways, particularly cricothyrotomy, are infrequently performed in the emergency department, with reported incidences of approximately 0.28% [8-10]. When instantaneous rigor involves the temporomandibular joint, it manifests as trismus and restricts mouth opening, rendering bag-mask ventilation, supraglottic airway insertion, and orotracheal intubation extremely difficult or impossible [1,11]. This low procedural frequency of emergency cricothyrotomy poses challenges for skill acquisition and retention. Evidence suggests that technical skill degradation may begin as early as one month and becomes significant after three months without practice [12,13]. Despite its rarity, emergency cricothyrotomy remains a critical life-saving intervention, underscoring the importance of regular high-fidelity simulation-based training [13].

In this case, instantaneous rigor affecting the muscles of mastication resulted in severe trismus immediately following cardiac arrest, necessitating emergency FONA via surgical cricothyrotomy. Timely recognition and decisive airway management were essential components of the resuscitative effort [13].

## Conclusions

Instantaneous rigor is a rare but clinically significant phenomenon that can abruptly compromise airway management during cardiac arrest. Emergency physicians should be aware of this entity and distinguish it from rigor mortis. Maintaining procedural competence in surgical airway techniques is essential to ensure timely airway control in such high-stakes situations.
